# A Rare Case of Ampullary Carcinoma with Complete Duodenal Obstruction as the Initial Manifestation

**DOI:** 10.70352/scrj.cr.25-0020

**Published:** 2025-04-16

**Authors:** Yuta Kakizaki, Akefumi Sato, Yoshitaka Enomoto

**Affiliations:** 1 Division of Surgery, Hiraka General Hospital, Yokote, Akita, Japan; 2 Division of Gastrointestinal Surgery, Iwate Prefectural Central Hospital, Morioka, Iwate, Japan

**Keywords:** ampullary carcinoma, duodenal obstruction, hyperbilirubinemia

## Abstract

**INTRODUCTION:**

Obstructive jaundice is often the first symptom of ampullary carcinoma, with a straightforward preoperative diagnosis. We report a rare case of ampullary carcinoma without jaundice as the initial symptom.

**CASE PRESENTATION:**

A 53-year-old man was admitted with abdominal distension and recurrent vomiting. Esophagogastroduodenoscopy revealed a complete duodenal obstruction without malignant findings. Computed tomography revealed wall thickening in the second portion of the duodenum. The common bile duct and main pancreatic duct were not dilated. As there was no evidence of malignancy, we performed gastrojejunostomy as a bypass to improve the symptoms. Five months later, follow-up blood examinations showed elevated total bilirubin levels, and computed tomography revealed persistent thickening of the duodenal wall with exacerbated dilation of common bile duct and main pancreatic duct. Mucosal biopsies from the oral and anal sides of the stenosis revealed no malignancy. Due to a strong suspicion of malignant disease and difficulty in preoperative biliary drainage, we performed pancreatoduodenectomy. Pathological examination revealed mucinous adenocarcinoma with submucosal and subserosal invasion of the duodenum. We finally diagnosed this case as ampullary carcinoma.

**CONCLUSIONS:**

The possibility of malignancy should be considered even in cases of duodenal obstruction that have not been diagnosed as malignant after repeated close examination.

## Abbreviations

CA19-9
: carbohydrate antigen 19-9
CEA
: carcinoembryonic antigen
CT
: computed tomography
EUS
: endoscopic ultrasonography
MRI
: magnetic resonance imaging
PD
: pancreatoduodenectomy
T-Bil
: total bilirubin

## INTRODUCTION

In general, obstructive jaundice, weight loss, epigastric pain, and diabetes mellitus are often initial symptoms of ampullary carcinoma.^1,2)^ In many cases of ampullary carcinoma, cancer cells are exposed on the tumor surface. Preoperative diagnosis of malignant diseases is relatively easy. Here, we report a rare case of ampullary carcinoma with complete duodenal obstruction without jaundice as the initial manifestation. To our knowledge, there has been 1 Japanese case report of ampullary carcinoma arising from duodenal obstruction without jaundice.^3)^ We describe our patient in this case report and discuss why ampullary carcinoma does not cause obstructive jaundice as the first symptom.

## CASE PRESENTATION

**Fig. 1:**
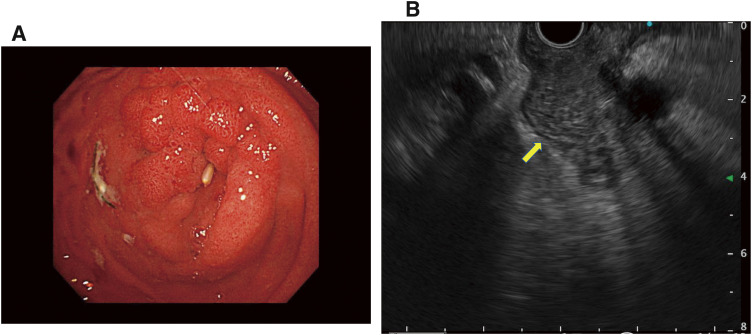
Esophagogastroduodenoscopy and endoscopic ultrasonography findings. (**A**) Esophagogastroduodenoscopy showed duodenal obstruction at the first visit. (**B**) Endoscopic ultrasonography showed wall thickening in the second portion of the duodenum. The yellow arrow showed the thickening duodenal wall.

**Fig. 2:**
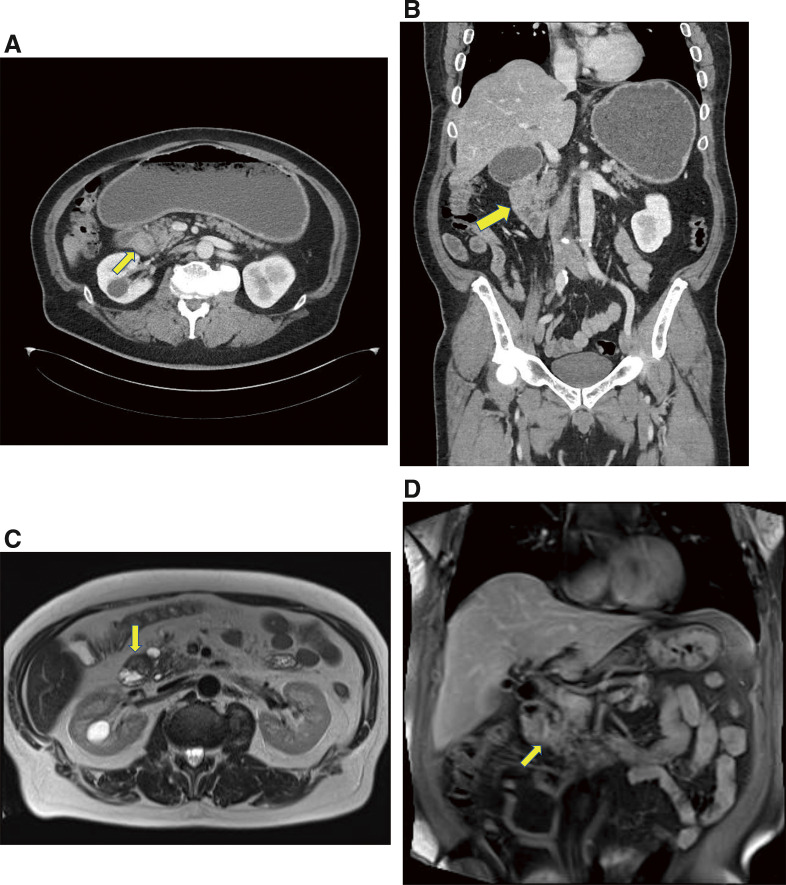
Computed tomography and magnetic resonance imaging findings at the first visit. (**A** and **B**) Computed tomography scan revealed duodenal obstruction and wall thickening in the second portion of the duodenum with stomach expansion, without lymph node swelling, and without dilation of the intrahepatic bile duct and main pancreatic duct (arrows). (**C** and **D**) Magnetic resonance imaging showed duodenal wall thickening (arrows).

A 53-year-old man presented to our hospital with abdominal distension and recurrent vomiting. His medical history included only hypertension, with no family history of disease. Esophagogastroduodenoscopy revealed complete obstruction of the second portion of the duodenum, which was covered by mucosa (**Fig. 1A**). Two biopsies revealed no malignant findings. Laboratory tests showed no elevation of carcinoembryonic antigen (CEA) or carbohydrate antigen (CA19-9), but small increases in DuPAN-2 (888 U/mL, reference range: ~150 U/mL) and SPAN-1 (69.3 U/mL, reference range: ~30 U/mL) levels. Computed tomography (CT) revealed duodenal obstruction due to wall thickening of the second portion of the duodenum with stomach expansion, but without lymph node swelling, dilation of the intrahepatic bile duct, or main pancreatic duct (**Figs. 2A** and **2B**). Endoscopic ultrasonography (EUS) detected thickening of the second portion of the duodenum, while extraduodenal and submucosal lesions were ruled out (**Fig. 1B**). Magnetic resonance imaging (MRI) confirmed duodenal wall thickening (**Figs. 2C** and **2D**).

At this time, we suspected atypical benign diseases, such as thickening of the duodenal muscle layer. Since there was no evidence of malignancy, we performed gastrojejunostomy with cholecystectomy as a bypass to improve symptoms induced by duodenal obstruction. During this surgery, we biopsied the serosa from the second portion of the duodenum, but the results showed an absence of malignancy.

**Fig. 3:**
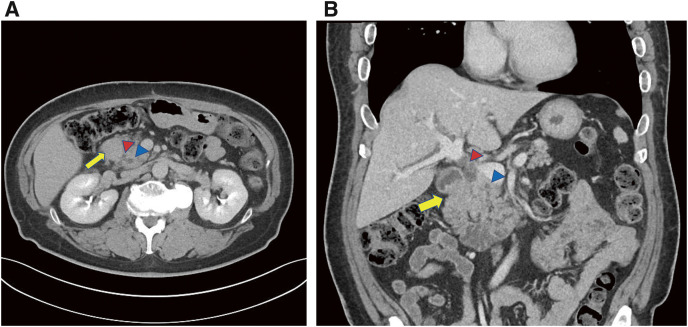
Computed tomography scan results at obstructive jaundice. (**A** and **B**) Computed tomography scan at readmission revealed duodenal wall thickening in the second portion of the duodenum (arrows) and dilation of the common bile duct (red arrowhead) and main pancreatic duct (blue arrowhead).

**Fig. 4:**
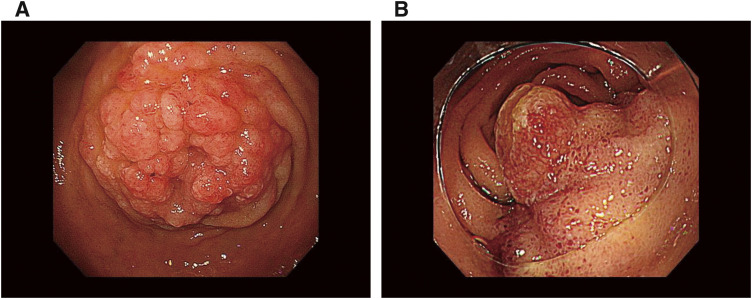
Esophagogastroduodenoscopy and balloon-assisted endoscopy findings at obstructive jaundice. Esophagogastroduodenoscopy at readmission detected duodenal obstruction on the pyloric side (**A**), and balloon-assisted endoscopy showed edematous mucosa on the anal side of the duodenum, while the papilla of Vater could not be identified (**B**).

Five months later, follow-up blood examinations revealed elevated levels of total bilirubin (T-Bil), amylase, and lipase. The tumor markers CEA and CA19-9 were not elevated, but DuPAN-2 and SPAN-1 levels were elevated (3969 and 266 U/mL, respectively). CT revealed wall thickening of the second portion of the duodenum and dilation of the common bile duct and main pancreatic duct (**Figs. 3A** and **3B**). Esophagogastroduodenoscopy detected duodenal obstruction on the pyloric side (**Fig. 4A**) and balloon-assisted endoscopy showed edematous mucosa on the anal side of the duodenum, while the papilla of Vater could not be identified (**Fig. 4B**). A histological examination conducted at the same time revealed no malignancy. Although jaundice worsened to 16.3 mg/dL of T-Bil, there was only slight dilation of the intrahepatic bile duct. We strongly suspected malignancy of the duodenum, pancreatic head, and distal bile duct.

**Fig. 5:**
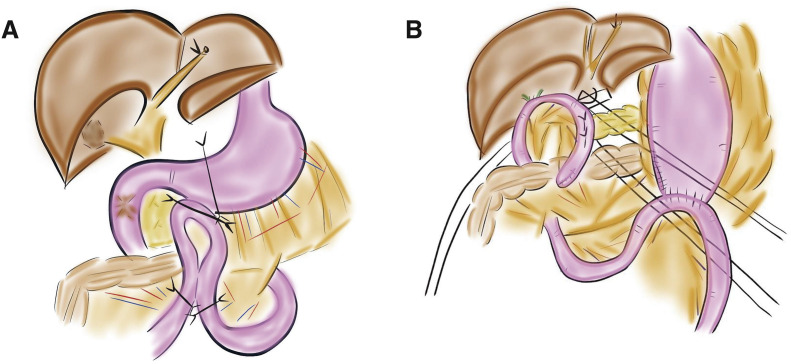
Operative procedures at second surgery. (**A**) The jejunum of the gastrojejunostomy was dissected. The stomach antrum was then dissected. (**B**) We performed pancreatoduodenectomy with regional lymph node dissection. There was no variation in vascular anatomy and no vascular invasion by the tumor.

We performed pancreatoduodenectomy (PD) without preoperative biliary drainage because of its difficulty. Initially, a laparotomy was performed, and the jejunum of the gastrojejunostomy was dissected. The stomach antrum was then dissected. We performed PD with regional lymph node dissection (**Figs. 5A** and **5B**). The operative time was 409 minutes, and the blood loss was 933 mL. Adhesions due to the first surgery and obesity (body mass index: 31) increased the blood loss. There was no variation in vascular anatomy and no vascular invasion of the tumor. The postoperative course was uneventful, and the patient was discharged 18 days after surgery. No recurrence has been observed at 24 months after surgery.

**Fig. 6:**
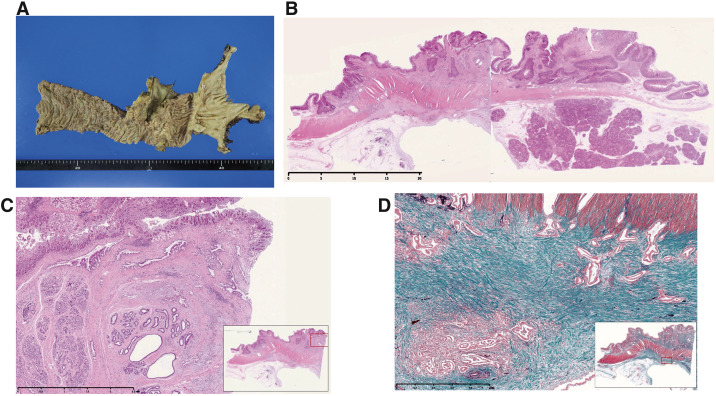
Gross and histological examination results. (**A**) Gross findings of the specimen. The tumor was located in the second portion of the duodenum and had resulted in circular wall thickening (60 × 63 × 27 mm). (**B** and **C**) Microscopic findings of hematoxylin–eosin stain showed mucinous adenocarcinoma with invasion inside and outside of the sphincter of Oddi, as well as submucosal and subserosal invasion. (**D**) Masson staining of elastica revealed adenocarcinoma invasion under the mucosa with a high degree of fibrosis.

In the resected specimen, the tumor was located in the second portion of the duodenum and had resulted in circular wall thickening (60 × 63 × 27 mm; **Fig. 6A**). Pathological examination revealed mucinous adenocarcinoma with invasion of the inside and outside of the sphincter of Oddi, submucosal and subserosal invasion, and 1 metastatic lymph node. The patient was finally diagnosed with ampullary carcinoma (AcApAb, non-exposed protruded type, 60 × 63 × 27 mm, moderately well > poorly mucinous carcinoma, T3b [Panc], pPV0, pPA0, INFb, Ly1b, V1a, Pn0, pN1 [#13a 1/3], cM0. HM0, PM0, EM0, and R0) (**Figs. 6B**–**6D**).

## DISCUSSION

Here, we report a rare case of complete duodenal obstruction caused by ampullary carcinoma, followed by obstructive jaundice. Since no evidence of malignancy existed, the patient could not have been diagnosed preoperatively. We performed PD without preoperative biliary drainage. Duodenal obstruction as the first symptom of biliary tract malignancies is very rare. To our knowledge, only a single case has been reported in the Japanese biomedical literature.^3)^

The ampulla of Vater is a complex region composed of 3 pathohistologically distinct anatomic structures: the common bile duct, pancreatic duct, and the duodenum, which adjoin the papilla of Vater. Ampullary carcinoma is a rare malignant disease, occurring in approximately 0.2% of all gastrointestinal tumors.^4)^ When comparing the subtypes of ampullary carcinoma in terms of pathology, they differ in prognosis with 5-year survival rates ranging from 20% for the pancreatobiliary subtype to 88% for the intestinal subtype in resected patients.^5)^ Furthermore, Adsay et al. reported that 249 ampullary carcinoma cases from 1469 PD specimens have been analyzed and clinicopathologically classified into 4 distinct subtypes based on the origin location: (1) intra-ampullary (25%), (2) ampullary-ductal (15%), (3) peri-ampullary-duodenal (5%), (4) ampullary carcinomas—not otherwise specified (“papilla of Vater”; 55%).^6)^ The feature of the ampullary-ductal cases represented carcinomas arising from non-tumoral (flat) intraepithelial neoplasms of the ducts and showed pancreatobiliary lineage, which constitutes the vast majority of pancreatobiliary subtype in the ampullary ductal cases (86%).^5,6)^ These tumors formed constrictive, sclerotic, plaque-like thickening of the walls of the common bile duct and/or pancreatic duct, resulting in mucosa-covered, button-like elevations of the papilla into the duodenal lumen. On the other hand, these had a low incidence of lymph node metastasis (41%).^6)^ Thus, it is not surprising that these tumors exhibit aggressive clinical behavior and the worst prognosis (3-year survival, 41%).^2,6)^ Despite having the worst survival among the ampullary carcinomas, patients with ampullary ductal cases still fared better than those with pancreatic ductal carcinoma (3-year survival, 11%) and distal common bile duct carcinoma (3-year survival, 29%).^6,7)^ Based on these features, we diagnosed this case as an ampullary-ductal subtype. The reason was the epicenter of the carcinoma in the ampullary common duct; its feature of invasion into surrounding tissue of the common bile duct and duodenum rather than spreading into the ductal lumen; and its tendency for intense fibrosis. In this case, it was considered that the cancer invaded mainly the duodenal stroma and the tissues surrounding the terminal end of the common bile duct with a high degree of fibrosis, causing duodenal obstruction without obstruction of the common bile duct. Therefore, repeated biopsies of the duodenal mucosa around stenosis showed no malignant findings.

Initially, malignant tumors, benign duodenal tumors, and duodenal muscle layer thickening were considered as differential diagnoses. In general, the differential diagnoses of duodenal obstruction in adults commonly include duodenal neoplasms (benign and malignant; primary or secondary), duodenal malformation/deformity, pancreatic pathology (severe acute or chronic pancreatitis, pancreatic pseudocysts, and pancreatic neoplasms), hepatobiliary pathology (neoplasms and Bouveret syndrome), postbulbar peptic ulcer disease, Crohn’s disease, retroperitoneal disease, thickened muscle layer, cholecystitis, compression by tumors or surrounding organs (e.g., aneurysm and superior mesenteric artery syndrome), and pancreatic malformation (e.g., annular pancreas).^8–10)^ The patient described here was an adult man with no medical history, and congenital disease was also ruled out. Results of preoperative CT and MRI were negative for duodenal diverticulum, compression by surrounding tissue, or the presence of a tumor, and there was no evidence of an annular pancreas. Positron emission tomography was not available preoperatively because of patient factors, and EUS was performed; however fine-needle aspiration was difficult because it was difficult to observe the frontal view. Since we suspected atypical benign diseases, such as thickening of the duodenal muscle layer, a gastrojejunal bypass was performed. There was no inflammation in the gallbladder, no abnormality on the duodenal serosal surface, and no malignant findings in the biopsy. However, because malignant disease could not be ruled out, the patient had to be followed up at short intervals after the first surgery.

In this case, the mucosal or serosal surfaces were not exposed to cancer, and a diagnosis could not be made before PD. When the patient visited our hospital, duodenal obstruction had already occurred, and repeated biopsies revealed no malignancy. It was difficult to perform EUS-guided fine-needle aspiration and endoscopic retrograde cholangiography to biopsy bile duct epithelium. After performing gastrojejunostomy as a bypass, balloon-assisted endoscopy showed edematous mucosa on the anal side of the duodenum while the papilla of Vater could not be identified. Biopsies from the oral and anal sides of the papilla of Vater revealed no malignancy. Since there had not been any exposure on the mucosal surface, we could not diagnose malignancy preoperatively. Furthermore, the tumor was located in the second part of the duodenum, surrounding the papilla of Vater, and partial resection of the duodenum was difficult. In this case positron emission tomography was not available preoperatively because of patient factors. Thus, no malignant findings were obtained, and a gastrojejunal bypass had to be selected for the first surgery. At that time, we should have strongly suspected malignancy and aggressively considered PD. In similar cases, positron emission tomography should be considered to obtain malignant findings.

We performed PD, the patient exhibited hyperbilirubinemia during surgery, and the postoperative course was uneventful. The patient displayed 16 mg/dL of T-Bil immediately before PD but was judged to be difficult to treat with endoscopic retrograde biliary drainage because of duodenal stenosis and an unidentified Vater papilla. The patient was also difficult to treat with percutaneous transhepatic biliary drainage because of slight intrahepatic bile duct dilatation. Furthermore, if the patient had biliary tract cancer, percutaneous transhepatic biliary drainage would have been undesirable because of the possibility of peritoneal dissemination of cancer. Performing PD without biliary drainage in patients with severe jaundice is controversial. Severe hyperbilirubinemia (arbitrarily defined in the literature but typically ~15 mg/dL) has been associated with hepatic synthetic, cardiac, and renal dysfunction as well as immune incompetence that may lead to a decline in performance status.^11–16)^ This patient had severe jaundice but no coagulopathy or organ dysfunction; therefore, we determined that the patient was operable. Postoperatively, there was a pancreatic fistula and a biochemical leak,^16)^ but no biliary infection was observed.

## CONCLUSION

We performed PD in a case of complete duodenal obstruction where the initial symptoms were caused by ampullary carcinoma, followed by obstructive jaundice. In cases of duodenal obstruction where no obvious cause can be identified, the presence of malignant disease may be suspected.

## ACKNOWLEDGMENTS

The authors would like to thank Dr. Yasuyuki Hara for writing assistance.

## DECLARATIONS

### Funding

No sources of funding for the research reported should be declared.

### Authors’ contributions

YK wrote the draft.

YK, AS, and YE were involved in the clinical management of the patient.

The final manuscript was read and approved by all authors.

All authors agree to be responsible for all aspects of the study.

### Availability of data and materials

All data generated or analyzed during this study are included in the published article.

### Ethics approval and consent to participate

This study was performed in accordance with the ethical standards of the 1964 Declaration of Helsinki and its subsequent amendments. This work does not require ethical considerations or approval.

### Consent for publication

Written informed consent was obtained from the patient for publication of this case report and accompanying images.

### Competing interests

The authors declare that they have no competing interest.
